# Supplementary material for: Experiences of health providers regarding implementation of the physiologic birth program in Iran: A qualitative content analysis
Source: PLoS One. 2023 Jun 30;18(6):e0283022. doi: 10.1371/journal.pone.0283022 (PMC10313046; doi:10.1371/journal.pone.0283022)
Supplement: Attachment — Submitted filename: Response to reviewer.docx [file pone.0283022.s002.docx]

**Response to reviewer’s comments**

Dear Editor

Re: Manuscript

Greetings and Regards

Please find attached a revised version of our manuscript “*[Experiences of health providers regarding implementation of the physiologic birth program in Iran: A qualitative content analysis*]”, after careful assessment of editor and reviewers' comments [*PLOS ONE*].

Your comments and those of the reviewers were highly insightful and enabled us to greatly improve the quality of our manuscript. In the following pages please see our point-by-point responses to each of the comments of editor and the reviewers.

We hope that the revisions in the manuscript and our accompanying responses will be sufficient to make our manuscript suitable for publication in [*PLOS ONE*]*.*

Yours sincerely,

Prof Parvin Abedi

Midwifery Department

Ahvaz Jundishapur University of Medical Sciences

**1. Please ensure that your manuscript meets PLOS ONE's style requirements, including those for file naming. The PLOS ONE style templates can be found at**

**https://journals.plos.org/plosone/s/file?id=wjVg/PLOSOne_formatting_sample_main_body.pdf and**

<https://journals.plos.org/plosone/s/file?id=ba62/PLOSOne_formatting_sample_title_authors_affiliations.pdf>

Thank you. All requirements of journal was checked and corrected in the paper.

**2. Thank you for stating the following financial disclosure:**

“**The funders had no role in study design, data collection and analysis, decision to publish, or preparation of the manuscript.”**

**At this time, please address the following queries:**

**a)        Please clarify the sources of funding (financial or material support) for your study. List the grants or organizations that supported your study, including funding received from your institution.**

**b)        State what role the funders took in the study. If the funders had no role in your study, please state: “The funders had no role in study design, data collection and analysis, decision to publish, or preparation of the manuscript.”**

These statements were added to the manuscript and cover letter.

c)        **If any authors received a salary from any of your funders, please state which authors and which funders.**

**d)        If you did not receive any funding for this study, please state: “The authors received no specific funding for this work.”**

**Please include your amended statements within your cover letter; we will change the online submission form on your behalf.**

Thank you. Added.

**3. Thank you for stating the following in the Acknowledgments Section of your manuscript:**

**“The expenses of this study were provided by Ahvaz Jundishapur University of Medical Sciences. The funder did not play any role in design, data collection, data interpretation, writing and submitting the manuscript to a journal.”**

**We note that you have provided additional information within the Acknowledgements Section that is not currently declared in your Funding Statement. Please note that funding information should not appear in the Acknowledgments section or other areas of your manuscript. We will only publish funding information present in the Funding Statement section of the online submission form.**

**Please remove any funding-related text from the manuscript and let us know how you would like to update your Funding Statement. Currently, your Funding Statement reads as follows:**

**“The funders had no role in study design, data collection and analysis, decision to publish, or preparation of the manuscript.”**

**Please include your amended statements within your cover letter; we will change the online submission form on your behalf.**

Thank you. Our statement about funding is same as above and we included it in the cover letter.

**4. In your Data Availability statement, you have not specified where the minimal data set underlying the results described in your manuscript can be found. PLOS defines a study's minimal data set as the underlying data used to reach the conclusions drawn in the manuscript and any additional data required to replicate the reported study findings in their entirety. All PLOS journals require that the minimal data set be made fully available. For more information about our data policy, please see** [**http://journals.plos.org/plosone/s/data-availability**](http://journals.plos.org/plosone/s/data-availability)**.**

**Upon re-submitting your revised manuscript, please upload your study’s minimal underlying data set as either Supporting Information files or to a stable, public repository and include the relevant URLs, DOIs, or accession numbers within your revised cover letter. For a list of acceptable repositories, please see http://journals.plos.org/plosone/s/data-availability#loc-recommended-repositories. Any potentially identifying patient information must be fully anonymized.**

**Important: If there are ethical or legal restrictions to sharing your data publicly, please explain these restrictions in detail. Please see our guidelines for more information on what we consider unacceptable restrictions to publicly sharing data: http://journals.plos.org/plosone/s/data-availability#loc-unacceptable-data-access-restrictions. Note that it is not acceptable for the authors to be the sole named individuals responsible for ensuring data access.**

**We will update your Data Availability statement to reflect the information you provide in your cover letter.**

Thank you. The following statement was added to the manuscript:

In order to preserve participants' confidentiality, and according to the requirements of the METC Groningen in which anonymity of participants must be guaranteed, we are not willing to share the qualitative datasets (the interview transcripts) in the main paper or additional supporting files. We cannot share the data due to ethical restrictions that the data contains potentially identifiable and sensitive information of the participants. Although we did remove personal identifiers from the interview transcripts (e.g. names and addresses), the transcripts are likely to contain references to the contextual identifiers in individual stories and make individuals identifiable. When providing their informed consent to participate in the study, participants were ensured their privacy would be protected. They did not provide consent for their data to be shared in a repository. We can provide access to the transcripts and audit trail on request and subject to certain conditions. Data requests must be addressed to the Reproductive Health Promotion Research Center of Ahvaz Jundishapur University of Medical Sciences, that will provide access after evaluating requests: [RHPRC@ajums.ac.ir](mailto:RHPRC@ajums.ac.ir).

**5. Please review your reference list to ensure that it is complete and correct. If you have cited papers that have been retracted, please include the rationale for doing so in the manuscript text, or remove these references and replace them with relevant current references. Any changes to the reference list should be mentioned in the rebuttal letter that accompanies your revised manuscript. If you need to cite a retracted article, indicate the article’s retracted status in the References list and also include a citation and full reference for the retraction notice.**

Thank you. The references were reviewed and none of them were retracted or had a correction.

**Additional Editor Comments to the Author:**

**Dear authors,
Thank you for submitting the manuscript to PLOS ONE. This manuscript is dealing with an important subject. Therefore, to improve it and receive deserved attention from the readers, some revisions are needed.**

- ***Response****: Thank you*

**Introduction
Measures stated in the first three lines of page 6 are related to making childbirth a satisfactory process and are not necessary physiologic birth. So, with this distinction in mind, PLS provide a precise definition of the physiologic birth.**

- *Response: [*it was added and highlighted, page 6]

**PLS clarify if the midwives working in a hospital are allowed to serve simultaneously as a private midwife and as personnel on duty on the same shift.**

- *Response: [*it was explained and highlighted it]

**PLS describe the impact of the COVID-19 pandemic on the holding of childbirth preparation classes in the country at the time of the study**.

- *Response: [*The impact of the COVID-19 epidemic on childbirth preparation classes was mentioned in the introduction. *Due to the fact that at the time of conducting this study, the COVID 19 pandemic in Iran was almost controlled by nationwide vaccination and that face-to-face physiologic childbirth classes were being implemented, this study did not have any limitations in this regard.*]

**Methods**

**PLS describe the rationale for the recruitment method. (Interview vs. focus group discussion)**

- *Response: [*It was added.

*Due to the maximum variety of the participants who were from different levels of service providers, including managerial, executive, clinical, and educational levels in both public and private sectors, the interviews were expected to provide a deep understanding of the phenomenon of physiological childbirth from the participants’ point of view.]*

**How many people refused to participate or dropped out? Reasons?**

- *Response: [No participant was excluded from the study.]*

**How many data coders coded the data?**

- *Response: [The coding process was performed by two authors (AM, PA).]*

**Results
 PLS provide a description of the coding tree.**

- *Response: [*Added]

**Discussion
 PLS discuss the generalizability of the results.**

- *Response: [*Added]

**PLS explain the probable effects of the COVID-19 pandemic on your results.**

- *Response: [*Added]

**PLS check the text especially the results section and quotes for grammatical errors**

- *Response: [*The manuscript was edited by a person who is expert in English literature]

**PLS use the COREQ checklist, or other relevant checklists listed by the Equator Network, to ensure complete reporting of the study and attach the completed checklist after revising the manuscript.**

- *Response: [*Done.]

**PLS discuss limitations and potential sources of bias.**

*Response: [There was no limitation in the implementation of this study.]*

**Reviewer Comments to Author**

**Reviewer: 1**

**1-It is better to mention the subcategories in the abstract**

- *Response: [Added]*

**2-In the obtaining consent form section, write both the consent to participate in the study and the consent to record the interviews were obtained.**

- *Response: [*Thank you. Added.]

**3-Please mention what was your strategy in case you were not allowed to record the interviews?**

- *Response: [The participants’ consent for recording the interview was obtained, and in case they did not allow the recording, field notes were taken.]*

**4-In purposive sampling, you should mention the purposive sampling with maximum variation in term of some variable such as duration of experience, .....**

- *Response: [Purposive sampling was done considering maximum variety in terms of the participants’ workplace, work experience (years), educational attainment, and age.]*

**5-Please mention the fully characteristics of the people participating in the research after each meaning unit. Ex (p10, 32y, midwife)**

- *Response:* [*Done]*

**6-You can merge the categories in one theme**

- *Response: [Considering the importance and breadth of the category, we preferred to keep the division in two themes.]*

**7- The subcategory entitled: Supervision, monitoring and continuous evaluation of the quality of childbirth preparation and physiologic birth classes is too long, please change it**

***Response***: [Done*.]*

**8-in subcategory” Assessing program implementation”, Can't you use the word evaluation instead assessing???please explain your reasons**

*Thank you. The word "assessing" was replaced by evaluation.*

**9- participants did not mention the effect of COVID-19 on the quality of participation of mothers in classes?**

- ***Response***: *[ The participants mentioned the problems of the online classes of physiological childbirth, which were categorized in the category of low quality of the classes*.*]*

**Reviewer: 2**

**In abstract: The year and place of the study should be stated**

- ***Response****: [*Added.*]*
